# The Role and Regulatory Network of the CiaRH Two-Component System in Streptococcal Species

**DOI:** 10.3389/fmicb.2021.693858

**Published:** 2021-07-14

**Authors:** Li-Yuan He, Yao-Jin Le, Zhong Guo, Sha Li, Xiao-Yan Yang

**Affiliations:** ^1^Zhuhai Key Laboratory of Basic and Applied Research in Chinese Medicine, Department of Bioengineering, Zhuhai Campus of Zunyi Medical University, Zhuhai, China; ^2^Center for Biological Science and Technology, Beijing Normal University, Zhuhai, China

**Keywords:** two-component system CiaRH, histidine kinase CiaH, response regulator CiaR, streptococci, regulatory network

## Abstract

Pathogenic streptococcal species are responsible for a broad spectrum of human diseases ranging from non-invasive and localized infections to more aggressive and life-threatening diseases, which cause great economic losses worldwide. Streptococci possess a dozen two-component systems (TCSs) that play important roles in the response to different environmental changes and adjust the expression of multiple genes to successfully colonize and infect host cells. In this review, we discuss the progress in the study of a conserved TCS named CiaRH in pathogenic or opportunistic streptococci including *Streptococcus pneumoniae*, *Streptococcus pyogenes*, *Streptococcus agalactiae*, *Streptococcus mutans*, *Streptococcus gordonii*, *Streptococcus sanguinis*, and *Streptococcus suis*, focusing on the function and regulatory networks of CiaRH, which will provide a promising strategy for the exploration of novel antistreptococcal therapies. This review highlights the important role of CiaRH and provides an important basis for the development of antistreptococcal drugs and vaccines.

## Introduction

*Streptococcus* is a genus of Gram-positive bacteria that can colonize humans and animals, and is the dominant species in the host oral cavity and upper respiratory tract (Patenge et al., 2013). Most streptococci are non-pathogenic and live in benign and commensal relationships with their hosts. However, several of them are pathogenic including *Streptococcus pneumoniae*, *Streptococcus pyogenes* (also known as group A *Streptococcus*, GAS), *Streptococcus agalactiae* (also known as group B *Streptococcus*, GBS), *Streptococcus mutans*, *Streptococcus gordonii*, *Streptococcus sanguinis*, and *Streptococcus suis*, which can cause a wide range of infectious diseases, such as pneumonia, meningitis, septicemia, scarlet fever, toxic shock, protophilic, endocarditis, dental caries, and streptococcal toxic shock syndrome (van der Poll and Opal, 2009; Ralph and Carapetis, 2013; Jiang et al., 2019). These pathogenic streptococcal species are responsible for substantial mortality and morbidity and pose a major threat to human health.

Streptococci have evolved several mechanisms to respond to environmental changes allowing bacterial survival and infection of host cells. One of them, two-component systems (TCSs), is widely distributed in bacteria and is found in some archaea, fungi, plants, and lower eukaryotes; however, they are absent in humans and other mammals (Wuichet et al., 2010; Capra and Laub, 2012; Galperin et al., 2018). In bacteria, a typical TCS consists of two different proteins: a histidine kinase (HK) and a cognate response regulator (RR). First, the HK receives external stimuli and autophosphorylates at a histidine (His) residue. Then, the phosphoryl group is transferred from the His to an aspartic acid (Asp) residue of the RR. Finally, the RR is activated by this phosphorylation and triggers an appropriate cellular response by interacting with the regulatory regions of the related target genes (Gao and Stock, 2009). Most bacteria possess more than 10 TCSs that regulate multiple cellular functions such as metabolism, virulence, stress response, biofilm formation, antibiotic resistance, and competence. Therefore, TCSs are considered promising targets for the development of novel antibacterial drugs (Gotoh et al., 2010).

Based on literature report and the P2CS (prokaryotic TCSs) database^1^, *S. pneumoniae* TIGR4 and R6 encode 13 TCSs (Throup et al., 2000); *S. mutans* UA159, *S. sanguinis* SK36, and *S. pyogenes* MGAS5005 contain 14 TCSs (Smith and Spatafora, 2012; Buckley et al., 2018); both *S. gordonii* str. Challis substr. CH1 and *S. suis* 2 possess 15 TCSs (Zheng et al., 2018); and *S. agalactiae* 2603V/R possesses 17 TCSs (Tettelin et al., 2002). Moreover, there is strain-to-strain variation in the number of TCS in these streptococcal species, for example, *S. agalactiae* 2603V/R possesses 17 TCSs, while *S. agalactiae* SA20-06 possesses 13 TCSs. One of the best characterized TCSs present in many streptococcal species is CiaRH, which is composed of the HK CiaH and the RR CiaR. In this review, we present an overview of the function and regulatory network of CiaRH in streptococci.

### The Homology of CiaRH in Streptococci

**FIGURE 1:**
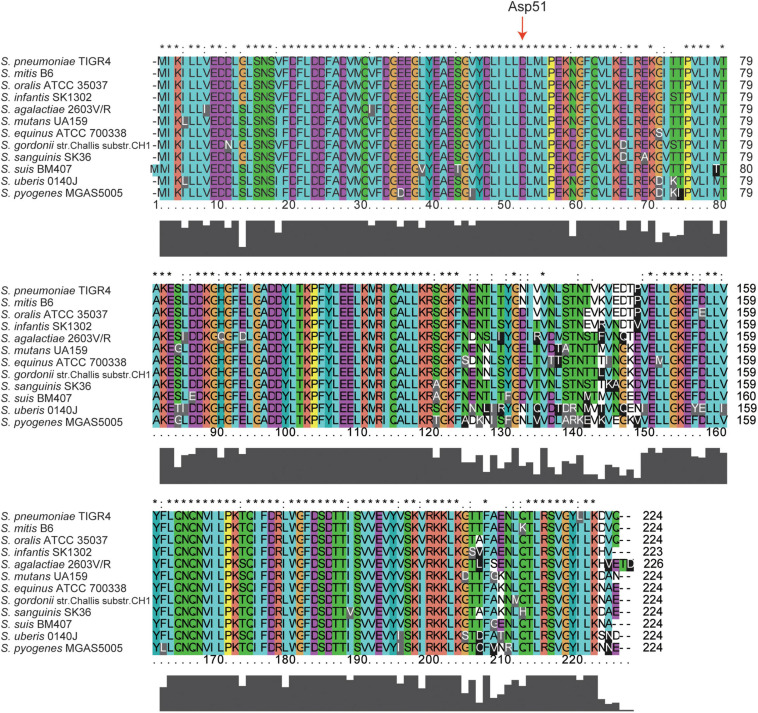
Multiple amino acid sequence alignment of CiaR across 12 streptococcal species. Homologous sequences with 91–100% similarities. The 12 streptococcal species, namely, *S. pneumoniae* TIGR4, *S. mitis* B6, *S. oralis* ATCC 35037, *S. infantis* SK1302, *S. agalactiae* 2603V/R, *S. mutans* UA159, *S. equinus* ATCC 700338, *S. gordonii* str. Challis substr. CH1, *S. sanguinis* SK36, *S. suis* BM407, *S. uberis* 0140J, and *S. pyogenes* MGAS5005. The residues labeled with * represent the invariant residues. The height in the bar graph represents the conservation of amino acids.

Amino acid sequence alignment analysis revealed that CiaH proteins share between 49 and 97% identical residues and between 66 and 98% similar residues (Table 1). CiaR proteins are much more conserved in streptococci, and the amino acid identities of CiaR proteins range from 85 to 99%, while amino acid similarities range from 91 to 100% (Table 1). These data suggested that CiaRH systems, especially CiaR proteins, are highly conserved in streptococci. Furthermore, almost all Asp residues (D) including Asp51, which receives the phosphoryl group from the His of CiaH (Guenzi et al., 1994), are invariant in all streptococcal CiaR proteins (Figure 1). Collectively, these data indicated that the CiaRH system, especially the CiaR protein, is highly conserved in streptococcal species.

**TABLE 1:** Amino acid identities and similarities of CiaR and CiaH proteins of various streptococci to *S. pneumoniae* TIGR4 CiaR and CiaH proteins.

**Bacteria**	**Identities with CiaR**	**Similarities with CiaR**	**Identities with CiaH**	**Similarities with CiaH**
*S. pneumoniae* TIGR4/R6/D39	100%/100%/100%	100%/100%/100%	100%/100%/100%	100%/100%/100%
*S. mutans* UA159/NN2025/LJ23	88%/88%/88%	92%/92%/92%	56%/56%/56%	73%/73%/73%
*S. gordonii* str. Challis substr. CH1/BCA22/DD07	90%/90%/91%	95%/95%/95%	66%/66%/66%	80%/80%/80%
*S. sanguinis* SK36/SK49/SK355	89%/89%/88%	94%/94%/95%	61%/60%/60%	73%/74%/72%
*S. pyogenes* MGAS5005/10270/10750	85%/85%/85%	91%/91%/91%	51%/51%51%	71%/71%/71%
*S. agalactiae* 2603V/R/H36B/A909	88%/88%/88%	95%/95%/95%	52%/52%/52%	72%/72%/72%
*S. suis* BM407/SC84/05HAS68	88%/88%/88%	94%/94%/94%	49%/49%/49%	66%/66%/66%
*Streptococcus mitis* B6/S022-V7-A3/ATCC 6249	99%/99%/97%	100%/100%/99%	97%/95%/84%	98%/98%/93%
*Streptococcus infantis* SK1302/SPAR10/ATCC 700779	94%/94%/94%	97%/97%/97%	88%/81%/81%	94%/89%/89%
*Streptococcus uberis* 0140J/NCTC3858/4672	85%/85%/85%	91%/91%/91%	51%/51%/50%	69%/70%/69%
*Streptococcus oralis* ATCC 35037/subsp. Tigurinus 1366/subsp. Tigurinus AZ_3a	98%/98%/97%	99%/99%/99%	84%/84%84%	93%/92%93%
*Streptococcus equinus* ATCC 700338/NCTC8140/10386	87%/88%/86%	93%/92%/92%	51%/52%/52%	69%/68%/68%

### Phosphorylation of the CiaRH System

Under a variety of growth conditions, the CiaRH system appears to be constitutively active in wild-type streptococci. Both CiaH and CiaR proteins contain a two-domain structure: a receiver domain and an effector domain. Commonly, CiaH senses environmental stimuli *via* receiver domain and autophosphorylates the appropriate His residue in its effector domain after binding ATP, subsequently transferring the phosphoryl group to a conserved Asp51 in the receiver domain of CiaR, thus activating CiaR to elicit a cellular response by changing the downstream gene expression (Guenzi et al., 1994). Although CiaH is necessary for sensing most signals, not all Cia input signals are CiaH-dependent, and CiaR can obtain phosphoryl groups independently of CiaH (Marx et al., 2014).

In *S. pneumoniae*, depending on the growth medium, deletion of *ciaH* weakly elicited the CiaR-dependent promoter activities or strongly inhibited them, suggesting that CiaH possesses kinase or phosphatase activities to phosphorylate or dephosphorylate CiaR, leading to the reversible regulation of gene expression (Halfmann et al., 2011). In the absence of CiaH, CiaR appears to be highly active (Halfmann et al., 2011). A subsequent study found that in the absence of CiaH, CiaR can obtain a phosphoryl group from acetyl phosphate (AcP) to become activated (Marx et al., 2014). Additionally, AckA, an AcP synthesis-related acetate kinase, can bind to CiaR and negatively regulate the AcP-dependent phosphorylation of CiaR (Marx et al., 2014; Kaiser et al., 2020). In addition to TCS kinases, a great number of proteins have been reported to control RRs, and the phosphorylation of serine/threonine/tyrosine and even the acetylation of RRs also affect RR-mediated regulation (Mitrophanov and Groisman, 2008; Kalantari et al., 2015; Janczarek et al., 2018). In addition to the phosphoryl groups donated by CiaH and AcP to CiaR, there may be other phosphodonors for CiaR, other phosphoreceptors for CiaH, or other post-translational modifications such as phosphorylation of serine/threonine/tyrosine residues of CiaR, which warrants further exploration.

## Major Functions of CiaRH in Streptococci

### Competence Development

In bacteria, competence is a transient physiological state that permits cells to uptake and integrates exogenous DNA into the bacterial genome, which plays a key role in virulence, biofilm formation, and antibiotic resistance (Claverys et al., 2006; Charpentier et al., 2012). Natural competence was originally discovered in *S. pneumoniae* and was later demonstrated in many bacteria such as in *S. mutans* but also in *S. suis* (Avery et al., 1979; Perry and Kuramitsu, 1981; Claverys et al., 2006). In streptococci, natural competence for genetic transformation can be stimulated by competence-stimulating peptide (CSP) or sigX-inducing peptide (XIP) and initiated by the com locus (Cheng et al., 1997; Alloing et al., 1998; Shanker et al., 2016). In Mitis and Anginosus *Streptococcus* groups, CSP is the product of the *comC* gene and is exported by the ComAB transporter; after that, extracellular CSP is sensed by the ComDE TCS (e.g., *S. pneumoniae*) (Havarstein et al., 1996; Cheng et al., 1997; Alloing et al., 1998; Shanker et al., 2016). In contrast, in all other groups of *Streptococcus* including *S. mutans*, *S. pyogenes*, *S. agalactiae*, and *S. suis*, XIP is encoded by *comS*, transported by the oligopeptide permease (Opp) transporter, and sensed by ComR to induce the expression of *sigX*, then initiates the competence (Mashburn-Warren et al., 2010; Shanker et al., 2016).

In *S. pneumoniae*, several studies have suggested that CiaRH negatively regulates competence development (Guenzi et al., 1994; Zahner et al., 1996; Echenique et al., 2000; Dagkessamanskaia et al., 2004). The *ciaH*_*C*306_ mutant represents CiaRH in the constitutively active state, which leads to a competence deficiency in *S. pneumoniae*; moreover, the addition of exogenous cannot stimulate the competence of the *ciaH*_*C*306_ mutant (Guenzi et al., 1994; Zahner et al., 1996). Echenique et al. (2000) found that *ciaRH* negatively regulates *comCDE* transcription and then modulates the competence stimulated by O_2_. Conversely, the inactivation of *ciaR* leads to competence induction. In addition, CiaRH is a pre-requisite for discontinuing the competent state of cells dependent on CiaR (Dagkessamanskaia et al., 2004). Interestingly, data on the contribution of CiaH and CiaR to competence are different. In *S. mutans*, CiaRH has been shown to be involved in the competence process, and competence development is influenced by CiaH but not CiaR (Qi et al., 2004; Ahn et al., 2006).

### Biofilm Formation

Biofilms are structured communities composed of one or multiple bacterial species that elicit many human infectious diseases, including dental caries (Maier, 2021). Twenty-five species of oral streptococci inhabit the human oral cavity, accounting for approximately 20% of the total oral bacteria (Nicolas and Lavoie, 2011). Dental caries is a classic biofilm-associated condition that is induced by diet (sugars) and microbiota–matrix interactions that occur on tooth surfaces, resulting in destruction of mineralized tooth tissue (Bowen et al., 2018; Chen et al., 2019). Among these oral streptococci, *S. mutans* is considered the most important cariogenic bacteria contributing to the formation of dental caries, and *S. sanguinis* and *S. gordonii* have been proposed to initiate oral biofilm formation (Kolenbrander et al., 2010; Bowen et al., 2018).

In contrast to the *ciaRH* operon in *S. pneumoniae*, the *ciaRH* operon in *S. mutans* contains three genes–*ciaR*, *ciaH*, and *ciaX*–that comprise a three-component signal transduction system (He et al., 2008). The CiaX contains a calcium-binding domain and *ciaXRH* operon expression is repressed by calcium through CiaX, and the inactivation of *ciaH*, *ciaR*, or *ciaX* results in attenuated biofilm formation (Qi et al., 2004; He et al., 2008). In addition to *S. mutans*, the CiaRH system and its cognate signal play a key role in biofilm development in other oral streptococci, e.g., *S. sanguinis* and *S. gordonii* (Kolenbrander et al., 2010; Davey et al., 2016a; Ota et al., 2018; Zhu et al., 2018). Biofilm formation and expression of CiaRH in thiol-disulfide oxidoreductase (SdbA)-deficient mutants are drastically increased, and further experiments validated that the effect of SdbA on biofilm formation is controlled by the CiaRH and ComDE TCSs in *S. gordonii* (Davey et al., 2016a,b).

CiaRH has also been proposed to be the key regulator of biofilm formation in *S. sanguinis* (Zhu B. et al., 2017; Ota et al., 2018; Zhu et al., 2018). Biofilms in *ciaR* deletion mutants are fragile, and the transcription of arginine biosynthesis genes (*argR*, *argB*, *argC*, *argG*, *argH*, and *argJ*) is increased (Zhu B. et al., 2017). Double deletion of *ciaR* and *argB* restored the biofilm formation ability that was lost in the *ciaR* mutant, indicating that *ciaR* influences biofilm formation by regulating an arginine biosynthesis pathway, especially regulation of the *argB* gene (Zhu B. et al., 2017). Moreover, CiaRH negatively regulated the expression of the type IV pilus retraction ATPase PilT and biofilm formation by controlling csRNA1-1 and csRNA1-2, two of the six known cia-dependent small RNAs (csRNAs) in *S. sanguinis* (Ota et al., 2018). While CiaR is regulated by the transcription factor *brpL*, mutation of *brpL* activates CiaR, which results in increased glucan production, cell aggregation, and biofilm formation (Zhu et al., 2018).

Similar effects of CiaRH on biofilm formation were also found in *S. pneumoniae* and *S. pyogenes*. Deletion of *ciaR/H* significantly decreased biofilm formation *in vivo*, which resulted in the inhibition of nasopharynx colonization by *S. pneumoniae* (Blanchette-Cain et al., 2013). In *S. pyogenes*, transcriptome analysis revealed that the superantigen SpeA suppresses biofilm formation, and this process is mediated by the CiaRH TCS (Babbar et al., 2019). Doubtlessly, inactivation of CiaRH can attenuate the biofilm-forming capacity in streptococci; therefore, targeting CiaRH may be an antibiofilm strategy for the development of antistreptococcal therapies.

### Antibiotic Resistance and Stress Tolerance

Antibiotic resistance in bacteria is increasing worldwide and becoming a serious threat to global human health (Doernberg et al., 2017; Doi et al., 2017). Many TCSs play crucial roles in antibiotic resistance by regulating genes to modify the antibiotic or its target, and biosynthesize efflux pumps to extrude the antibiotic (Huang et al., 2020). Competence and biofilm development are implicated in antibiotic resistance, and CiaRH regulates competence and biofilm development processes. Therefore, CiaRH also indirectly participates in antibiotic resistance.

In *S. pneumoniae*, CiaRH was originally reported to be involved in cefotaxime susceptibility, and the *ciaH*_*C*306_ mutant was resistant to cefotaxime (Guenzi et al., 1994; Giammarinaro et al., 1999). Currently, it is known that CiaRH also participates in cycloserine, bacitracin, vancomycin, and penicillin resistance (Haas et al., 2005; Mascher et al., 2006; Rogers et al., 2007; El Khoury et al., 2017). cDNA microarray analysis revealed that vancomycin can induce the expression of CiaRH in *S. pneumoniae* TIGR4 (a vancomycin-sensitive strain) but not in Tupelo (a vancomycin-tolerant strain) (Haas et al., 2005). Furthermore, CiaRH in the active state results in resistance to cycloserine, bacitracin, and vancomycin, while bacteria with CiaRH in the inactive state are hypersusceptible to these antibiotics (Mascher et al., 2006). Both DNA microarrays and transcriptome analysis revealed that CiaRH was induced when *S. pneumoniae* strains were exposed to penicillin (Rogers et al., 2007; El Khoury et al., 2017).

Much less is known about the contribution of CiaRH to antibiotic resistance in oral streptococci. To colonize and thrive on teeth, *S. mutans* expresses a set of genes to resist several antibacterial factors, including cationic antimicrobial peptides (AMPs) derived from the host. A report by Mazda et al. (2012) described that *ciaRH* regulated the teichoic acid biosynthesis operon *dlt* to resist AMPs in *S. mutans* biofilm cells.

In addition to participating in antibiotic resistance, CiaRH also has a central role in the regulation of stress tolerance, such as oxidative stress and acid tolerance (Ibrahim et al., 2004; Cortes et al., 2015). In the absence of *ciaR*, *S. pneumoniae* was more sensitive to oxidative stress, and this phenomenon was restored by complementation with high-temperature requirement A protein (HtrA) (Ibrahim et al., 2004). Cortes et al. (2015) provided evidence that CiaRH and ComDE TCSs participate in acid tolerance to help pneumococcus survive in acidic environments (e.g., in pneumocytes).

Likewise, several reports revealed that CiaRH is associated with acid tolerance in *S. mutans* (Qi et al., 2004; Zhu W. et al., 2017). The *ciaH* gene greatly reduced the growth rate of *S. mutans* under acidic culture conditions (Qi et al., 2004). A recent study revealed that sRNA133474 (a small non-coding RNA) negatively regulated the mRNA expression of *ciaR*, *liaR*, and *covR* to contribute to the acid tolerance of *S. mutans* in dental caries lesions (Zhu W. et al., 2017). The CiaRH of *S. gordonii* also contributes to acid tolerance, and the growth of *ciaRH*, *comDE*, or *vicRK* mutants was slower than that of the wild-type strain under acidified media (Liu and Burne, 2009).

In *S. agalactiae*, Δ*ciaR* mutant exhibited a lower survival rate than the wild-type strain when exposed to cationic AMPs, lysozyme, and ROS (Quach et al., 2009). In *S. suis*, DNA microarray data showed that acidic stress conditions can stimulate the expression of *ciaR/H* genes, indicating that CiaRH may play an important role in protecting *S. suis* against acidic stress or in adapting to these conditions (Yan et al., 2011).

In *S. pyogenes*, the natural competence is initiated by ComRS and SigX, but not by ComDE and CiaRH (Shanker et al., 2016). Thus, antibiotic resistance between *S. pyogenes* wild-type and *ciaH* mutant strains was not significantly different (Riani et al., 2007).

### Bacteriocin Production

Bacteriocins, proteins or peptides with pronounced antimicrobial activity, are produced by certain bacteria to inhibit the growth of neighboring bacteria belonging to the same or different species (Negash and Tsehai, 2020; Gradisteanu Pircalabioru et al., 2021). In *S. mutans*, *S. gordonii*, and *S. pneumoniae*, the CiaRH system and its cognate signal play a key role in bacteriocin production (Qi et al., 2004; Dawid et al., 2009; Davey et al., 2016b). Mutacins are bacteriocins produced by *S. mutans* that exhibit antimicrobial effects on closely related streptococcal species and on other bacteria in dental plaques (Hamada and Ooshima, 1975). The deletion of *ciaH*, but not of *ciaR* mutant, repressed mutacin I production (Qi et al., 2004). Interestingly, data on the contribution of CiaRH to bacteriocin production in *S. gordonii* and *S. pneumoniae* are in opposition to those in *S. mutans*. In *S. pneumoniae*, bacteriocin (also named pneumocin MN) production was negatively regulated by CiaH, deletion of *ciaH* induced the production of pneumocin MN, and activation of *ciaH* diminished the production of pneumocin MN (Dawid et al., 2009). Similarly, in *S. gordonii*, CiaRH was activated, while the production of bacteriocin was inhibited in the Δ*sdbA* mutant, and loss-of-function CiaRH in the Δ*sdbA* mutant could restore bacteriocin production (Davey et al., 2016b).

### Virulence and Pathogenesis

Over the past decade, a large number of studies have demonstrated that CiaRH also has important roles in the virulence and pathogenesis of streptococci. During the infection process, bacteria can adhere to, invade, and colonize the host organism with the help of virulence factors, leading to related infectious diseases. In *S. pneumoniae*, CiaRH controls the expression of *htrA*, which is connected to virulence. In a murine model of pneumonia, disruption of *ciaRH* or *htrA* significantly reduced pneumococcal colonization in nasopharyngeal tissue (Sebert et al., 2002). A further study showed that supplementation of the *S. pneumoniae* Δ*ciaR* mutant with HtrA can recover virulence (Ibrahim et al., 2004). In the process of colonization, sialic acids (Sias) serve as carbon sources for *S. pneumoniae* and are consumed by the sialidase NanA (Vimr et al., 2004). Recently, a study reported that CiaR sensed N-acetylneuraminic acid (Neu5Ac, the most abundant Sias in humans) to result in an increase in *htrA* and other genes involved in sialic acid metabolism and ROS tolerance, contributing to increased pneumococcal virulence (Hentrich et al., 2016). In addition, CiaR stimulated the P_*lic*1*P*1_ promoter activity of choline metabolism-related *lic* loci and promoted pneumococcal colonization in the host (Johnston et al., 2016).

*Streptococcus agalactiae* is a major opportunistic pathogen that asymptomatically colonizes the vaginal and gastrointestinal tracts of healthy human adults, including pregnant women; however, this species causes invasive diseases, including meningitis, pneumonia, and sepsis in newborns (Doran and Nizet, 2004). To cause meningitis, *S. agalactiae* must be able to invade and survive within brain microvascular endothelial cells and then breach the blood–brain barrier (BBB) to enter the central nervous system (CNS). CiaRH is regarded as a key factor involved in intracellular survival and the ultimate disease progression of meningitis. Indeed, *S. agalactiae* strains lacking the *ciaR* gene exhibited impaired survival in neutrophils, murine macrophages, and human brain microvascular endothelial cells (hBMECs), and a competitive infection assay in a mouse model demonstrated that the Δ*ciaR* mutant had a lower survival advantage in the bloodstream and brain than in the wild-type strain (Quach et al., 2009). Furthermore, mutants in CiaR-regulated genes *SAN_2180* and *SAN_0039* (Δ*2180* and Δ*0039*) were determined to have a lower intracellular survival rate in hBMECs and a lower survival advantage in the bloodstream and brain in a mouse infection model. These results observed in Δ*2180* and Δ*0039* were similar to those in Δ*ciaR*, implying that *S. agalactiae* may modulate *SAN_2180* and *SAN_0039* gene expression through CiaRH to promote intracellular survival and virulence (Mu et al., 2016). Recently, a study conducted by Spencer et al. (2019) compared the transcriptomes of *cas9* (CRISPR-associated protein-9) and *ciaR* mutants (Δ*cas9* and Δ*ciaR*) and determined that the RR CiaR was regulated by Cas9 to contribute to vaginal colonization and persistence of *S. agalactiae*.

*Streptococcus suis* is a highly invasive pathogen that causes infectious diseases in both swine and humans, resulting in manifestations such as meningitis, septicemia, pneumonia, endocarditis, arthritis, and septic shock (Lun et al., 2007; Wertheim et al., 2009). Li et al. (2011) reported that CiaRH contributes to the virulence of *S. suis*, and the *ciaRH* deletion mutant (Δ*ciaRH*) exhibited lower adherence to epithelial cells (Hep-2 and PIEC) and higher susceptibility toward killing by RAW2647 macrophages than the wild-type strain. Moreover, *in vivo*, Δ*ciaRH* was attenuated in murine and pig animal models of infection, but showed increased survival rates in mice and pigs and reduced bacterial loads in specific organs. Similarly, the Δ*ciaRH* strain also showed an increased survival rate in the zebrafish larval model compared with the wild-type strain (Zaccaria et al., 2016). Recently, because *ciaR* was significantly increased when *S. suis* was stimulated with human polymorphonuclear leukocytes (PMNs), CiaRH has been proposed to be a critical regulatory system for resistance to phagocytosis by human PMNs (Chang et al., 2018).

Overall, these data indicated that CiaRH potentially promotes virulence and pathogenesis including the survival rate, adherence, and colonization capabilities of *S. pneumoniae*, *S. agalactiae*, and *S. suis*. However, less is known about the virulence and pathogenesis of CiaRH in *S. mutans*, although it was shown that CiaRH was induced by *Candida albicans* to enhance the *S. mutans* biofilm accumulation, thereby regulating the virulence of *S. mutans* (He et al., 2017).

### Cell Wall Biosynthesis and Autolysis

Teichoic acid is an important component of the Gram-positive cell wall that plays vital roles in autolysis, drug resistance, biofilm formation, and virulence (Wu et al., 2020). The two operons *dltABCD* and *licABC* are engaged in teichoic acid biosynthesis and are required for D-alanylation and choline metabolism of teichoic acid in streptococci, respectively. Several studies have shown that CiaRH controls the expression of *dltABCD* and *licABC* in *S. pneumoniae* (Mascher et al., 2003; Dagkessamanskaia et al., 2004; Halfmann et al., 2007; Slager et al., 2019). Autolysis is a programmed cell death process triggered by autolysin that causes cell wall self-digestion. The CiaRH system in the defective state accelerated the autolysis of pneumococci, while the CiaRH system in the active state prevented autolysis (Giammarinaro et al., 1999; Lange et al., 1999). As mentioned above, activation of the CiaRH system resulted in resistance to cell wall inhibitors, while deletion of the CiaRH system resulted in hypersusceptibility to cell wall inhibitors. Taken together, these results suggest that the CiaRH system in *S. pneumoniae* induces the expression of *dltABCD* and *licABC* to confer resistance to cell wall inhibitors and autolysis.

In addition, a similar effect of CiaRH on *dltABCD* and autolysin was found in *S. mutans*, which also results in antibiotic resistance and stress tolerance (Wu et al., 2010; Mazda et al., 2012). However, whether CiaRH affects cell wall biosynthesis and autolysis in other streptococci remains to be tested.

### Regulatory Network of CiaRH in Streptococci

As mentioned above, the CiaRH TCS affects streptococcal competence development, biofilm formation, antibiotic resistance, stress tolerance, bacteriocin production, virulence, pathogenesis, cell wall biosynthesis, and autolysis. However, some biological functions of the CiaRH TCS in streptococci are the opposite, and the regulatory network of CiaRH is different among streptococcal species.

### Regulatory Network of CiaRH in *S. pneumoniae*

**FIGURE 2:**
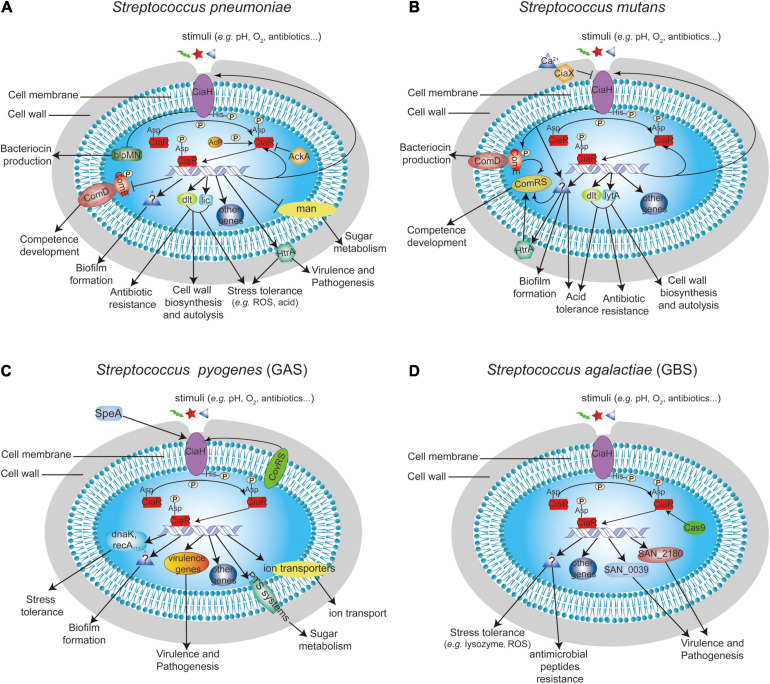
Summary of the known function of CiaRH and its regulatory network in **(A)** *S. pneumoniae*, **(B)** *S. mutans*, **(C)** *S. pyogenes*, and **(D)** *S. agalactiae*.

The global gene expression effect of CiaRH in *S. pneumoniae* has been reported in four genomic/profiling-related manuscripts. The authors of these studies demonstrated that CiaRH controls the expression of more than 70 genes, and most genes were positively regulated by CiaRH (Mascher et al., 2003; Dagkessamanskaia et al., 2004; Halfmann et al., 2007; Slager et al., 2019; Figure 2A). In 2003, a study conducted by Mascher et al. (2003) revealed that CiaRH regulates the mannose phosphotransferase system (*manLMN*), phosphorylcholine system (*licABC*), Acetyl esterase (*axe*), ABC transporter (*cyl*), amylomaltase and maltose phosphorylase (*malPM*), teichoic acid biosynthesis system (*dltABCD*), serine protease (*htrA-spo0J*), conserved hypothetical protein (*spr0782*), extracellular protein (*spr0931*) operons, and 39 genes from 20 competence regulons, including *comCDE*. In addition to these genes, CiaRH also controls the expression of the DNA biosynthesis-related gene *dnaA-dnaN* and TCS03 (*hk03-rr03*) in *S. pneumoniae* (Dagkessamanskaia et al., 2004). It is worth noting that CiaRH can affect the expression of *ciaRH*, thus forming a feedback loop (Dagkessamanskaia et al., 2004). A further work showed that CiaR can bind to TTTAAG-N5-TTTAAG, a repeat sequence in the promoters of regulated genes, and thereby directly regulate the expression levels of 24 genes from 15 promoters (Halfmann et al., 2007). Except for *manLMN*, which was negatively controlled by CiaR, 21 genes, including five small non-coding csRNAs (*ccnABCDE*), were positively regulated by CiaR, and these genes were associated with choline modification of teichoic acids, sugar metabolism, stress responses, chromosome segregation, and protease maturation (Halfmann et al., 2007). A recent transcriptome study demonstrated that 38 genes distributed over 18 operons were directly or indirectly regulated by CiaR (Slager et al., 2019). Among these genes, six translation-related genes (*rimP*, *nusA*, *SPV_0480*, *SPV_0481*, *infB*, and *rbfA*), 23S rRNA [uracil(1939)-C(5)]-methyltransferase (*rlmCD*), and a novel uncharacterized non-coding RNA (*srf-21*) were found to be CiaR-regulated genes for the first time (Slager et al., 2019).

### Regulatory Network of CiaRH in Oral Streptococci

The regulatory network of CiaRH has also been reported in oral streptococci, including *S. mutans*, *S. sanguinis*, and *S. gordonii*. A total of 100 genes encoded on the *S. mutans* chromosome were regulated by CiaRH, including its own gene *ciaXRH* (Wu et al., 2010; Figure 2B). Moreover, eight Cia regulons were identified, including the *ciaXRH* operon itself, which is directly regulated by CiaR. The consensus sequence upstream of the putative −10 regions of these genes where the CiaR protein was bound to the promoters was NTTAAG-n5-WTTAAG (Wu et al., 2010). In addition, CiaRH can lead to an increase in the expression of the teichoic acid biosynthesis operon *dlt* in *S. mutans* biofilm cells (Mazda et al., 2012).

In *S. sanguinis*, CiaRH exerts its regulatory effect by mediating the expression of arginine biosynthesis genes, arginine/histidine permease genes, and csRNAs (Zhu B. et al., 2017; Ota et al., 2018). Less is known about the regulatory network of CiaRH in *S. gordonii*. Loss of SdbA can activate the CiaRH system, thereby negatively regulating the Com system to reduce bacteriocin production (Davey et al., 2016b).

### Regulatory Network of CiaRH in *S. pyogenes*

*Streptococcus pyogenes* is an exclusive human pathogen that causes diseases ranging from pharyngitis, impetigo, abscesses, cellulitis, sepsis, necrotizing fasciitis, and streptococcal toxic shock syndrome to acute post-streptococcal glomerulonephritis, acute rheumatic fever, and rheumatic heart disease (Carapetis et al., 2005; Walker et al., 2014). In this bacterium, CiaRH was regulated by the CovRS TCS and was associated with virulence (Graham et al., 2002), and CiaRH sensed SpeA to modulate biofilm formation (Babbar et al., 2019; Figure 2C). In addition, a study conducted by Riani et al. (2007) established transcriptome analyses between the wild-type strain and the *ciaH* mutant, which revealed that CiaH influences the transcription of 132 genes (63 genes were upregulated and 69 genes were downregulated in the *ciaH* mutant compared with the wild-type strain), including genes that encode divalent cation and other ion transporters, PTS systems, ribosomal proteins, virulence proteins, stress response proteins, and hypothetical and phage proteins (Figure 2C). However, unlike CiaRH in *S. pneumoniae*, only six stress response genes of 132 genes were activated in the *ciaH* mutant of *S. pyogenes*, and no cell wall turnover genes showed changes between wild-type and *ciaH* mutant strains (Riani et al., 2007).

### Regulatory Network of CiaRH in *S. agalactiae*

The effect of CiaRH on bacterial virulence, survival, and colonization has been well studied in *S. agalactiae*. Microarray data coupled with quantitative RT-PCR data revealed that CiaR positively regulated the expression of several genes, including hypothetical protein (SAN_2180) and peptidases (SAN_0039, SAN_0058, and SAN_0059) (Quach et al., 2009). Further studies showed that the SAN_2180 and SAN_0039 proteins were modulated by CiaR to enhance the virulence of *S. agalactiae* in mice (Mu et al., 2016; Figure 2D). Moreover, CiaR was regulated by Cas9 and contributed to *S. agalactiae* vaginal colonization and persistence (Spencer et al., 2019; Figure 2D).

## Concluding Remarks and Future Perspectives

Multidrug-resistant bacteria, including multidrug-resistant streptococci, have increased in prevalence and become a major threat to global public health. Novel drugs with modes of action different from conventional antibiotics are urgently needed to combat bacterial infections caused by these pathogens. Because TCSs are widely distributed in bacteria but do not exist in humans, targeting TCSs has been explored as a novel strategy to combat bacterial infections, especially drug-resistant bacterial infections (Worthington et al., 2013; Hirakawa et al., 2020; Rajput et al., 2021). The CiaRH TCS composed of CiaH and CiaR is an important signal transduction system that mediates various streptococcal behaviors, including competence development, biofilm formation, antibiotic resistance, stress tolerance, bacteriocin production, virulence, pathogenesis, cell wall biosynthesis, and autolysis. Moreover, the CiaRH system, especially the CiaR protein, is highly conserved in streptococcal species. Therefore, CiaRH may serve as an attractive antibiofilm or antivirulence target to develop new drugs to fight against various drug-resistant streptococcal infections. However, several issues remain to be addressed, e.g., (a) in addition to the known functions, what other functions does CiaRH possess? (b) Can CiaH or CiaR serve as markers to screen cell wall antibiotic-resistant streptococci? (c) Which drugs can target CiaR for the treatment of streptococcal infections? A more complete understanding of the CiaRH system will aid in preventing and controlling streptococcal infections.

## Author Contributions

L-YH wrote the manuscript. Y-JL and ZG analyzed the data and provided the visual images. SL and X-YY provided the initial idea, funding, and edited the manuscript. All authors contributed to manuscript revision and read and approved the submitted version.

## Conflict of Interest

The authors declare that the research was conducted in the absence of any commercial or financial relationships that could be construed as a potential conflict of interest.

## Footnotes

1 http://www.p2cs.org/
